# Robotic-assisted pyeloplasty in a five-day-old infant with severely infected hydronephrosis: The very young case report and review of literature^☆^

**DOI:** 10.1016/j.ijscr.2025.111478

**Published:** 2025-06-09

**Authors:** Boshen Shu, Shufeng Zhang, Jian Gao, Lin Wang, Xiaohui Wang

**Affiliations:** Department of Pediatric Surgery, Henan Provincial People's Hospital, Zhengzhou 450003, Henan Province, China

**Keywords:** Hydronephrosis, Pyeloplasty, Robotic-assisted surgery, Antenatal hydronephrosis

## Abstract

**Introduction:**

Postnatal management of newborns with antenatal hydronephrosis remains controversy. Most of the newborns with antenatal hydronephrosis are asymptomatic with only rare ones who develop renal insufficiency. We described a pediatric robotic assisted pyeloplasty for treating a five-day-old infant, who was diagnosed as left-sided hydronephrosis with infection two months before birth.

**Case presentation:**

A Da Vinci robotic surgical system with three arms and 5 mm trocar was used, providing free rotation of the robotic arm with different degree and making the anastomosis easier. The surgery lasted for 235 min, was uneventful and successful. The infant received postoperative management, including anti-infection measures and nutritional support. The follow up renal ultrasonography at 4 months (12.7 mm) and 17 months (9.6 mm) indicated progressive reducing anteroposterior diameters of renal pelvis. The scar was satisfactory due to better cosmetic results of robotic-assisted pyeloplasty.

**Clinical discussion:**

Robotic pyeloplasty is becoming the standard of treating children hydronephrosis owning to its minimally invasive access, faster recovery time and better cosmetic results. This article reported a very young hydronephrosis case with infection and presented a brief review of former reports to improve the understanding of rare hydronephrosis caused by antenatal hydronephrosis, and its potential treatment managements.

**Conclusion:**

To our knowledge, this case is unique and may be the youngest reported instance worldwide via a Da Vinci robotic surgical system in a child with hydronephrosis, which enhances the safe applicability of robotic pyeloplasty in the very young infant.

## Highlights

• Postnatal management of newborns with antenatal hydronephrosis remains controversy.
• Most of the newborns with antenatal hydronephrosis are asymptomatic.
• The present case may be the youngest reported instance with hydronephrosis using robotic surgical system.
• Robotic surgery strengthens 3D magnification imaging and faster learning curve for the surgeon.
• This case fills a research gap in the quite young infants with hydronephrosis who undergo robotic surgery.

## 1 Introduction

Antenatal hydronephrosis (ANH), the fetal renal pelvis dilation, is a common condition which is observed in 1–4.5 % of all pregnancies [1]. The dilation of the fetal renal collecting system can concern either the renal pelvis alone or the dilation of both the pelvis and the calices. Most of newborns with ANH are completely asymptomatic at birth. Only rare newborns (< 5 % of infants with ANH) develop a renal insufficiency or even needs renal transplantation [2]. Therefore, it is important to distinguish children at risk and apply surgery if necessary.

Here, we presented a pediatric robotic pyeloplasty in a five-day-old newborn with severely infected hydronephrosis. Compared with the conventional laparoscopic surgery, robotic surgery strengthens numerous key points including 3D magnification imaging, jitter filtering function and faster learning curve [3]. To the best of our knowledge, the present case is unique and may be the youngest reported instance worldwide using a Da Vinci robotic surgical system in a child and performing an anastomosis between the distal ureters and renal pelvis wall, filling a research gap in the quite young infants with hydronephrosis who undergo robotic surgery. This case report has been reported in line with the SCARE checklist [4].

## 2 Case report

### 2.1 Patient information

A male infant was admitted to the hospital due to left-sided infected hydronephrosis secondary to congenital ureteropelvic junction obstruction (UPJO) found by prenatal examination two months before birth and had a constant crying. This child underwent preoperative ultrasound examination 2 days old and was classified as intermediate risk category. A marked stricture was observed at the junction between the renal pelvis and ureter.

### 2.2 Personal and family history

The child had no family history of reproductive and urinary disease.

### 2.3 Physical examination

The birth weight was 3650 g. A palpable mass in the abdomen can be found and the size was 30.0 mm × 10.2 mm × 10.8 mm. No other positive signs were detected.

### 2.4 Laboratory examination

Urine test revealed conductivity was 3.6 mS/cm, lower than the standard range (5–38mS/cm). Blood test revealed white blood cell was 11.95 ∗ 10^9^/L, within the standard range for newborns (9–30 ∗ 10^9^/L).

### 2.5 Imaging and ultrasound examination

**Fig. 1:**
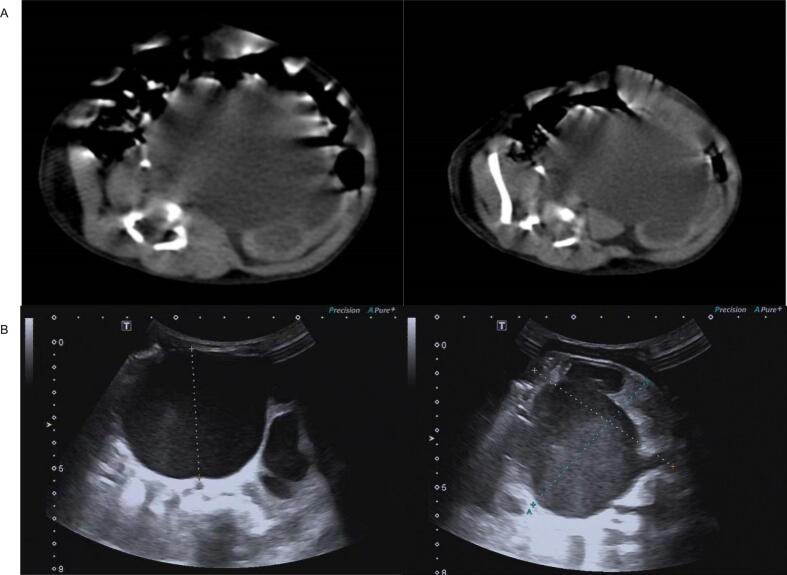
(A) Outcomes of preoperative CT indicated severe hydronephrosis and ureterectasis of left-sided moiety, along with left ureteral opening was dislocated; (B) Preoperative ultrasound examination indicated severe hydronephrosis.

Preoperative computerized tomography (CT) and ultrasound examination showed severe hydronephrosis and ureterectasis of left-sided moiety, along with left ureteral opening was dislocated (Fig. 1). The anteroposterior diameter of the renal pelvis was 51.2 mm, and the renal parenchymal thickness was 2.2 mm. No significant abnormalities were observed in the renal parenchyma echoes and the middle segments of the ureter. The distal segment demonstrated a cystic dilation projecting into the bladder lumen.

### 2.6 Robot-assisted surgery

**Fig. 2:**
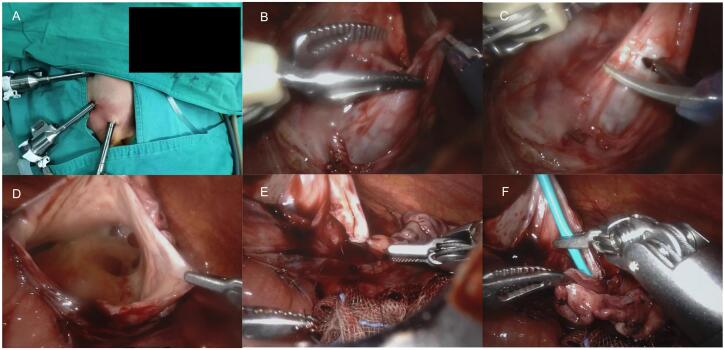
(A) Positions of the Da Vinci Trocar; (B) Identifying the stricture ureter; (C) Trimming excess renal pelvis; (D) Exposing the renal pelvis; (E) Making anastomostomosis of the renal pelvis valve and the lowest point of ureteral cut; (F) Inserting double J ureteral tubes with the assistance of guidewire.

Before surgery, we used ceftazidime via intravenous drip to prevent preoperative infection. Given that the unique characteristics of pediatric patients, we placed this child at the lateral edge of the table at a tilt of 30°. In the meantime, the patient was positioned with the lower limb in 90° knee flexion while maintaining full hip extension of the upper limb. The open Hassan technique was applied, and CO_2_ insufflation target was 8–10 mm Hg. After general anesthesia, we immobilized the child in a 30° left-side reclined position with the lumbar region elevated. We inserted trocars in the supraumbilical position, inverse McBurney point, and 3 cm below the xiphoid process respectively (Fig. 2A). The pneumoperitoneum was established with CO_2_ to 10 mm Hg and the eyepiece as well as forceps were used. The retrocolic access was applied to find the ureteropelvic junction (stenosis segment) and trim excess renal pelvis (Fig. 2B and C). And then we exposed the renal pelvis, as well as made anastomostomosis of the renal pelvis valve and the lowest point of ureteral cut with stitches (Fig. 2D and E). The following steps included indwelling double J ureteral tubes with the assistance of the guidewire (Fig. 2F). Next, a pararenal pelvis drainage tube was placed. Each instrument was withdrawn from the abdomen carefully and the normal structure of the umbilicus was rebuilt afterwards. Ceftazidime via intravenous drip was given to prevent infection and Vitamin K1 was used to prevent bleeding.

## 3 Results

**Fig. 3:**
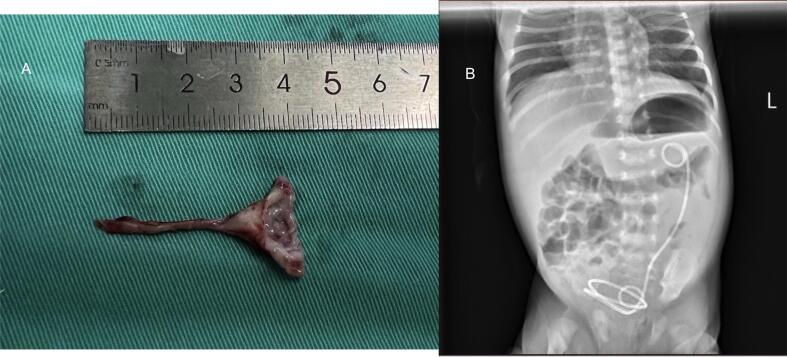
(A) Excess renal pelvis and the stricture ureter which were removed during the surgery; (B) Postoperative situation of the double J ureteral tubes.

The total operative time was 235 min and the surgery progressed successfully, no supplementary operative port placement or open procedure was required. After surgery, we applied ECG monitoring and box-type oxygen (3–5 L/min PRN) for the infant to maintain oxygen saturation. Excess renal pelvis and the stricture ureter which were removed during the surgery were shown in Fig. 3A. The X-ray was used to test the situation for double J ureteral tubes after surgery (Fig. 3B). The child was discharged home 6 days after surgery. The follow up renal ultrasonography at 4 months (12.7 mm) and 17 months (9.6 mm) indicated progressive reducing anteroposterior diameters of renal pelvis. The scar was satisfactory due to better cosmetic results of robotic-assisted pyeloplasty.

## 4 Discussion

To the best of our knowledge, this may be the youngest reported robotic pyeloplasty in a child with hydronephrosis worldwide using a Da Vinci robotic system. Minimally invasive surgery has been widely applied in pediatric surgery field and the research activity has increased over the last 30 years, with a golden decade in the early 21st century [5]. Furthermore, the introduction of robotic-assisted laparoscopic platform in pediatric surgery has its new horizons and advantages, such as decreasing the morbidity, accelerating postoperative recovery, more rapid learning curve for the surgeon, and improving cosmetic result, which is also a driving factor for us to use it [6].

A review of the published literature on pediatric robotic-assisted surgery revealed that, cases for children with low body weight or quite young age who underwent robotic-assisted surgery have been reported seldomly and there existed several controversial opinions regarding that robotic surgery was suitable for infants or not [7,8]. The controversy can be attributed to conducting the surgery in remarkably tiny spaces and its relative value in young infants, along with the influence of body weight on the result in children [1,9]. In this case, we successfully performed robotic pyeloplasty in a quite young child (5 days old) and found that adequate intestine preparation prior to surgery facilitated the exposure of surgical field obviously. Besides, the proper traction via sutures of the narrow ureter distal end and upper renal pelvis was beneficial for less aggressive manipulation of the tissue, which made flexible surgery work possible. We took continuous stitches as the strategy for reducing the occurrence of stenosis in the anastomosis and this proved to be feasible.

Several limitations about the application of robotic-assisted surgery in children should be mentioned. Firstly, the Da Vinci surgical system is more expensive for total hospitalization costs and longer installation time compared to conventional laparoscopy, though this cost differential may resolve with time and surgeons' technical skills. Secondly, a normalized robotic program of study or training protocol is still unavailable in pediatric surgery.

## 5 Conclusion

In summary, we reported a five-day-old case of severely infected hydronephrosis treated by robotic-assisted pyeloplasty successfully, to our knowledge, which may be the youngest reported instance worldwide. Infant hydronephrosis necessitates prompt recognition and initiation of surgical therapy to arrest severe urinary tract pathologies. This case enhances the safe applicability of robotic pyeloplasty in the very young infant. Nevertheless, robotic instruments developed particularly for pediatric surgery still have a long way to go and more infant cases with long-term follow-ups will be necessary for further validation.

## CRediT authorship contribution statement

Xiaohui Wang, Boshen Shu: Conceptualization; Xiaohui Wang: Resources; Jian Gao, Lin Wang: Data curation; Boshen Shu: Writing – original draft; Shufeng Zhang, Jian Gao, Lin Wang, Xiaohui Wang: Writing – review & editing.

## Ethical approval

Ethical approval was waived by the institution's Research Ethics Committee. Case reports involving a single patient encountered during routine clinical care are exempt from ethics approval at our institution.

## Patient perspective

The patient's family expressed relief and satisfaction with the appropriate surgical intervention and subsequent recovery.

## Research registration number

Not applicable.

## Funding sources

This study was supported.

## Patient consent for minors

Written informed consent was obtained from the patient's parents/legal guardian for publication and any accompanying images. A copy of the written consent is available for review by the Editor-in-Chief of this journal on request.

## Declaration of competing interest

The authors declare no conflict of interest.

## Data Availability

Data sharing not applicable to this article as no datasets were generated or analyzed during the current study.
